# Characterization of hepatic tumors using [^11^C]metomidate through positron emission tomography: comparison with [^11^C]acetate

**DOI:** 10.1186/2191-219X-3-13

**Published:** 2013-02-27

**Authors:** Anne Roivainen, Alexandru Naum, Heikki Nuutinen, Rauli Leino, Heimo Nurmi, Kjell Någren, Riitta Parkkola, Johanna Virtanen, Markku Kallajoki, Harry Kujari, Jari Ovaska, Peter Roberts, Marko Seppänen

**Affiliations:** 1 Turku PET Centre, Turku University Hospital, University of Turku, Kiinamyllynkatu 4-8, Turku 20521, Finland; 2 Center for Nuclear Medicine/PET, Department of Radiology, Haukeland University Hospital, Bergen 5021, Norway; 3 Department of Medicine, Turku University Hospital, University of Turku, Turku, 20521, Finland; 4 Department of Radiology, Turku University Hospital, University of Turku, Turku, 20521, Finland; 5 Department of Pathology, Turku University Hospital, University of Turku, Turku 20521, Finland; 6 Department of Gastroenterological Surgery, Turku University Hospital, University of Turku, Turku 20521, Finland; 7 Department of Nuclear Medicine, PET and Cyclotron Unit, Odense University Hospital, DK-5000, Odense C, Denmark

**Keywords:** [^11^C]acetate, Focal nodular hyperplasia, Hepatocellular carcinoma, Metastases, [^11^C]metomidate, Positron emission tomography

## Abstract

**Background:**

Using positron emission tomography (PET), we compared two tracers, [^11^C]metomidate ([^11^C]MTO) and [^11^C]acetate ([^11^C]ACE), for the characterization of hepatic tumors.

**Methods:**

Thirty-three patients underwent PET with [^11^C]MTO and [^11^C]ACE and magnetic resonance imaging (MRI). Based on the histology of the tumor biopsy, 14 patients had hepatocellular carcinoma (HCC), 9 patients had focal nodular hyperplasia (FNH), and 10 patients had other types of hepatic tumors. Tumor uptake was evaluated by calculating the maximum and mean standardized uptake value and tumor-to-liver ratio.

**Results:**

Altogether, 120 hepatic lesions (59 HCC, 18 FNH, 30 metastases of different primaries, 9 adenomas, and 4 regenerating nodules of liver cirrhosis) were detected by MRI. The overall tumor detection rate was slightly higher for [^11^C]MTO (39%) than for [^11^C]ACE (33%). [^11^C]ACE was more sensitive for HCC detection (50% versus 43%, respectively), whereas [^11^C]MTO was more sensitive for FNH detection (78% versus 44%, respectively). In HCC patients, the tumor grade correlated with [^11^C]ACE, but not with [^11^C]MTO. All of the patients with liver metastases, from various primary tumors (*n* = 10), were negative for both tracers.

**Conclusions:**

Due to low sensitivity, [^11^C]MTO and [^11^C]ACE PET have only limited value in diagnosing hepatic tumors.

## Background

Diagnostic assessment of primary liver tumors and metastases by various imaging modalities remains challenging. The challenge is in distinguishing between benign and malignant lesions, and often, a histopathological examination of a biopsy specimen is required for a definite diagnosis [1,2]. Noninvasive anatomic imaging modalities, including computed tomography (CT), magnetic resonance imaging (MRI), as well as ultrasound (US), are commonly used for the detection and characterization of intrahepatic processes [3-5]. Dynamic contrast-enhanced MRI facilitates the characterization of some liver tumors, for example, hemangioma, focal nodular hyperplasia (FNH), and typical hepatocellular carcinoma (HCC). However, the fundamental limitation of anatomical imaging, in general, is the limited capability of detecting malignant hepatic tumors [6,7].

In liver imaging, 2-[^18^F]-fluoro-2-deoxy-*D*-glucose ([^18^F]FDG) positron emission tomography (PET)/CT was proven to be highly sensitive in detecting hepatic metastases of different primary tumors and distant tumor spread [8-10]. However, the performance of [^18^F]FDG PET imaging in the detection of HCC is not optimal because of the variable tracer uptake which depends on tumor differentiation. PET with [^11^C]acetate ([^11^C]ACE) could serve as an alternative for the detection of HCC lesions, and the results by other authors have revealed high sensitivity and specificity for HCC [11-13]. In addition, [^11^C]ACE PET detects prostate cancer effectively [14,15]. Intravenously injected [^11^C]ACE enters the Krebs cycle as a substrate for β-oxidation in fatty acid synthesis and cholesterol synthesis. Fatty acid synthesis may be the major reason for the uptake of [^11^C]ACE by hepatic tumors [16].

Besides [^11^C]ACE, radiolabeled choline tracers ([^11^C]choline, [^18^F]fluorocholine) have also revealed high sensitivity for the diagnosis of HCC as well [17,18]. [^18^F]fluorocholine has been registered in several EU countries in this indication. However, the combination of two tracers, for example [^18^F]FDG and [^11^C]ACE or [^18^F]FDG and [^18^F]fluorocholine, improves the detection and staging of HCC especially [18,19]. Nevertheless, an ideal PET tracer capable of detecting all liver lesions does not exist, and thus, there is still room for the evaluation of other PET tracers in patients with HCC.

Metomidate (methyl derivative of etomidate) binds to gamma-aminobutyric acid (GABA) receptors and inhibits 11β-hydroxylase (CYP11B1, P45011β), an enzyme essential in the biosynthesis of aldosterone and cortisol. [^11^C]metomidate ([^11^C]MTO) was a PET tracer for the imaging of adrenocortical tumors [20,21]. The GABA receptor is upregulated in HCC, and it may be a potential target for [^11^C]MTO, which would promote [^11^C]MTO as a useful marker for HCC [22].

In this study, we tested the hypothesis if PET imaging with [^11^C]MTO could detect hepatic tumors. Accordingly, we examined [^11^C]MTO PET in patients with HCC, FNH, and other types of hepatic tumors in comparison with [^11^C]ACE PET imaging.

## Methods

### Subjects

This prospective study involved 33 patients (19 males and 14 females with mean age of 55 years, ranging from 16 to 77 years) with detected liver masses (CT/MRI/US) or metastases together with a known primary tumor. The study was carried out at Turku University Hospital, Turku, Finland. The patients had not been operated on or received any oncological treatment prior to inclusion. The study was conducted according to the guidelines of the Declaration of Helsinki, and the study protocol was approved by the Joint Commission of Ethics of Turku University Hospital. Each subject gave their written informed consent after the purpose, nature, and possible risks of the study had been explained to them.

The histological diagnosis was based on a percutaneous needle biopsy and/or surgical resection, except for five typical FNH lesions that were characterized and followed up by MRI. Of the 33 patients, 14 had HCC (one highly, nine moderately, and four poorly differentiated), 9 patients had FNH, 6 patients had liver metastases (four of colon carcinoma, one of a carcinoid tumor, and one of unknown origin), 2 patients had regenerating nodules of liver cirrhosis, 1 patient had cholangiocarcinoma, and 1 patient had hepatocellular adenoma. The patients were divided into the following groups according to the histology of the primary tumor: group I (14 patients with HCC), group II (9 patients with FNH), and group III (10 patients with other types of liver tumors).

### Magnetic resonance imaging

MR imaging was done on a 1.5 T scanner (Gyroscan Intera CV Nova Dual, Philips Medical Systems, Best, The Netherlands) using a sensitivity encoding body coil. The patients underwent a dynamic contrast-enhanced MRI, and this was used as the gold standard for measurement.

The MRI procedure consisted of coronal and axial balanced fast field echo sequences (echo time (TE) = 1.39 to 1.77 ms, repetition time (TR) = 2.78 to 4.12 ms, flip angle = 60°, and matrix = 224), T2-weighted axial fat saturation sequence (TE = 70 ms, TR = 1600 ms, flip angle = 90°, and matrix = 256), and T1-weighted axial in-opp and coronal in-phase sequences (TE = 2.3 and 4.6 ms, TR = 178 to 179 ms in axial and TR = 206 to 207 ms in coronal plane, flip angle = 80°, and matrix = 192). All sequences were scanned with 355- to 440-mm field of view and 7-mm slice thickness. T1-weighted coronal and axial sequences were repeated with intravenous gadolinium. The latter included 20, 60, and 180 s of dynamic contrast-enhanced imaging.

### PET scanning

All PET examinations were performed after an overnight fast. The production of positron-emitting tracers [^11^C]MTO and [^11^C]ACE has been described previously elsewhere [21,23]. An antecubital venous catheter was inserted in the right arm for the injection of tracers. PET imaging was performed either on a GE Advance PET (General Electric Medical Systems, Milwaukee, WI, USA; 11 patients) or an ECAT HR+ (Siemens/CTI, Knoxville, TN, USA; 22 patients) scanner with the patient in a supine position. All patients were studied with both tracers. For 11 patients, the [^11^C]MTO and [^11^C]ACE PET were done on the same day; for 13 patients, within 24 h; and for 10 patients, within 2 weeks. After the completion of a transmission scan (5 min using an externally rotating ^68^Ge/Ga rod), the subjects were given an intravenous bolus of 646 ± 108 MBq of [^11^C]MTO or 672 ± 73 MBq of [^11^C]ACE. A dynamic scan of the hepatic region, lasting 25 min (6 × 60 s, 3 × 180 s, and 2 × 300 s), was done to assess the radioactivity concentration as a function of time of the liver hotspots on the axial field of view. Following the dynamic acquisition, static emission images were acquired (25 to 45 min), covering the area from the level of the clavicle and moving down to the pelvic floor (2D mode, four to five bed positions, 5 min each). After the emission scanning, a 2-min transmission scan was performed at each table position.

All the data were corrected for dead time, radioactive decay, and photon attenuation. PET images were reconstructed using ordered-subset expectation maximization reconstruction algorithm and displayed in a 128 × 128 matrix, 35 or 47 transverse slices with a slice thickness of 4.5 mm. The final in-plane resolution in the reconstructed and Hann-filtered images was approximately 5 mm at 10 cm from the center of the gantry.

### MR image analysis

An image analysis of the MRI scans was performed in order to detect and locate the liver tumors accurately. The number and size of the lesions were measured. MRI analysis was also used to characterize the tumors prior to the final histological examination. Five out of nine FNH lesions were characterized and monitored by MRI. The MR images were analyzed by an experienced senior radiologist.

### PET image analysis

All PET images were reviewed on a computer screen in the transaxial, coronal, and sagittal planes along with maximum-intensity projection images. Two experienced reporters evaluated independently the tracers’ uptake both visually and semi-quantitatively. The degree of radiotracer uptake in the tumor was visually scored by comparing the apparent tumor to the surrounding liver parenchymal uptake as negative (i.e., less-than-liver or equivalent-to-liver uptake) or positive (i.e., higher-than-liver uptake). In case of discrepancies between the two reviewers, a joint reading was held to reach a consensus.

All PET images were fused with the corresponding MR images to ensure an accurate anatomic identification of the tumor. For a semi-quantitative analysis, a 15-mm circular region of interest (ROI) was drawn on three consecutive transaxial image planes covering the tumor. The maximum standardized uptake value (SUV_max_) and the mean SUV (SUV_mean_) of the lesion were calculated from the maximum/mean radioactivity concentration of the tumor ROIs between 25 to 45 min after injection. An ROI of the same size was used to evaluate the SUVs of the adjacent normal liver tissue in the same patient, and the tumor-to-liver (*T*/*L*) ratio was calculated by dividing the tumor SUV by the liver SUV. For patients who showed little or no lesion-related tracer uptake, the ROI was placed at the anatomical position of the lesion depicted on MR using direct visual alignment of both sets of images. If more than one lesion were positive, the lesion which showed the most intense uptake was chosen as representative of all lesions.

### Statistical analyses

Results are expressed as mean ± standard deviation (SD). Analysis of variance was used to compare the SUV_max_, SUV_mean_, and the *T*/*L* ratio among the three study groups. The SUV_max_ and SUV_mean_ parameters in tumors showing radiotracer uptake and those with no uptake were compared using an unpaired *t* test.

Correlations between HCC grade and tumor SUV_max_ or SUV_mean_ were calculated using Spearman’s rank correlation test. All statistical tests were performed with the SAS statistical analysis system (Enterprise Guide, version 2.0, SAS Institute Inc., Cary, NC, USA). A *P* value <0.05 was considered statistically significant.

## Results

### MRI findings

Altogether, 120 hepatic lesions (59 HCC, 18 FNH, 30 metastases of different primaries, 9 adenomas, and 4 regenerating nodules of liver cirrhosis) were detected by MRI. In group I, 2 patients had a unifocal tumor, and 12 had multifocal tumors. The mean tumor size of the largest lesion detected by MRI in each patient was 10.6 cm (range 3 to 19 cm). In group II, four patients had unifocal tumors, and multifocal tumors were in five patients; the mean tumor size of the largest FNH lesion was 4.8 cm (range 1.5 to 9.5 cm). In group III, nine patients had several tumor lesions, and one patient had a unifocal lesion. The mean tumor size of the metastatic lesions was 5.6 cm (range 0.5 to 11.6 cm).

### PET findings

**Figure 1:**
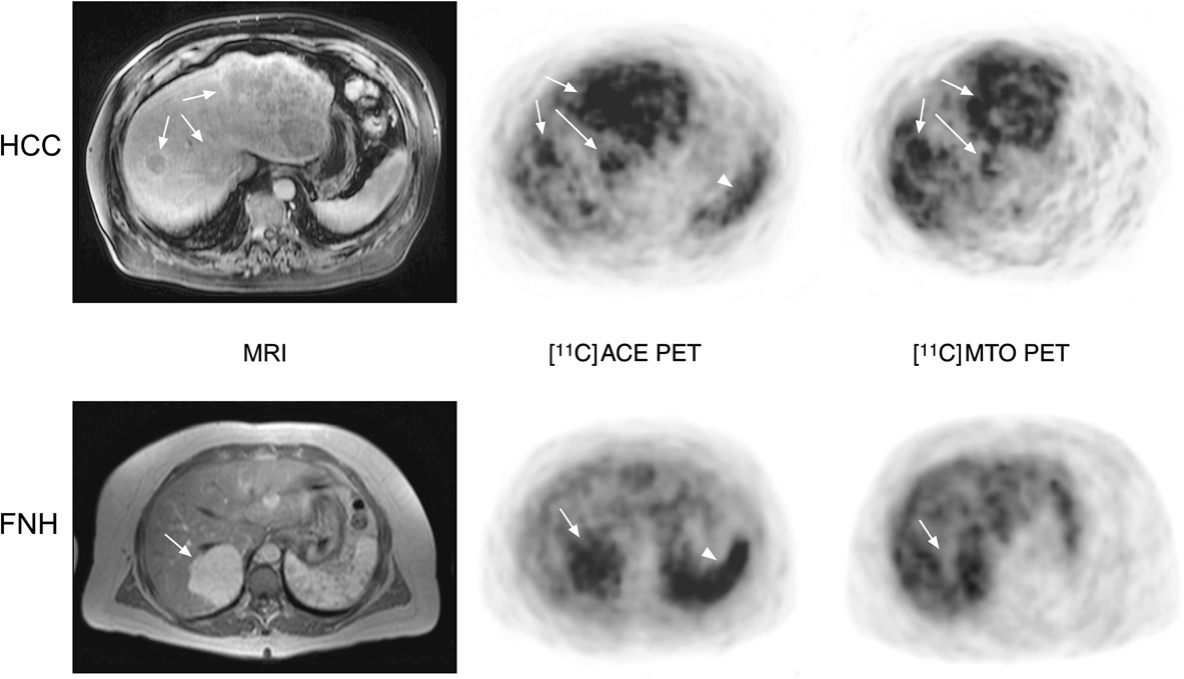
**Representative transaxial MRI and PET images of patients with hepatocellular carcinoma and focal nodular hyperplasia.** Arrows indicate tumors, and arrowheads indicate normal uptake in the spleen. HCC: A 71-year-old male had three tumors; the largest tumor (diameter, 3 cm) was biopsy-proven as a HCC grade II lesion. FNH: A 43-year-old female had biopsy-proven FNH.

Based on the visual assessment of the radiotracer uptake (Figure 1), on a per-patient basis, there were positive findings in 13/33 of the patients investigated with [^11^C]MTO PET and in 11/33 when [^11^C]ACE was used. Increased accumulation of both tracers was observed in 9/33 of cases, while in 18/33, neither of the tracers allowed the visualization of any of the tumors. Table 1 shows the results of the sensitivity and specificity analyses on a patient-per-patient basis for [^11^C]MTO and [^11^C]ACE PET imaging of hepatic tumors. Interestingly, group III, i.e., patients with types of tumors other than HCC or FNH, was negative for both tracers.

**Table 1:** **Sensitivity and specificity of [**^**11**^**C]MTO and [**^**11**^**C]ACE PET imaging of hepatic tumors based on patient-per-patient analysis**

**Group**	**Sensitivity (%)**		**Specificity (%)**	
	**[**^**11**^**C]ACE**	**[**^**11**^**C]MTO**	**[**^**11**^**C]ACE**	**[**^**11**^**C]MTO**
Group I (HCC, *n* = 14)	50	43	79	63
Group II (FNH, *n* = 9)	44	78	71	75
Group III (other liver tumors, *n* = 10)	NA	NA	NA	NA

NA, not applicable.

As regard the various tumor types, in group I, [^11^C]MTO detected 27/59 of the HCC lesions, 26/59 by [^11^C]ACE, and both tracers detected 22/59. When both tracers were positive, [^11^C]ACE detected more lesions compared to [^11^C]MTO (three patients with [^11^C]ACE; one patient with [^11^C]MTO). In group 2, [^11^C]MTO detected 9/18 of the FNH lesions, 5/18 by [^11^C]ACE, and both detected 4/18. The whole-body [^11^C]MTO PET imaging identified 14 unknown distant metastases in four patients, while [^11^C]ACE PET demonstrated 11 distant metastases in three patients, all in the HCC patient group.

The summary, tumor SUV_max_, SUV_mean_, and *T*/*L* ratio values (acquired between 35 to 40 min post-injection) for each of the study groups are shown in Table 2. Patients with FNH lesions (group II) had a significantly different *T*/*L* ratio from those with miscellaneous types of liver tumors (group III) when studied with [^11^C]MTO (*P* = 0.002). For [^11^C]ACE, the *T*/*L* ratio was also significantly different between groups I and III (*P* = 0.007). In patients with HCC (group I), significant negative correlations existed between tumor grade and SUV_max_ (*R*^2^ = −0.66, *P* = 0.010) and SUV_mean_ (*R*^2^ = −0.54, *P* = 0.046) for [^11^C]ACE.

**Table 2:** **Tumor uptake of [**^**11**^**C]ACE and [**^**11**^**C]MTO in different study groups**

**Group**	**[**^**11**^**C]ACE**			**[**^**11**^**C]MTO**		
	**SUV**_**max**_	**SUV**_**mean**_	***T*/*L***	**SUV**_**max**_	**SUV**_**mean**_	***T*/*L***
Group I (HCC; *n* = 14)	8.17 ± 3.03	5.68 ± 2.06	1.44 ± 0.75^*^	9.84 ± 6.36	6.43 ± 4.68	1.19 ± 0.81
Group II (FNH; *n* = 9)	7.36 ± 3.51	5.27 ± 2.44^*^	1.25 ± 0.31	12.82 ± 5.23	9.93 ± 3.61	1.51 ± 0.32^*^
Group III (other liver tumors; *n* = 10)	5.67 ± 3.16	4.21 ± 2.48	0.75 ± 0.28	7.76 ± 4.62	5.78 ± 3.90	0.75 ± 0.39

Results are expressed as mean ± SD. SUV, standardized uptake value; *T*/*L*, tumor-to-liver ratio. ^*^P < 0.05 versus group III (unpaired *t* test).

**Figure 2:**
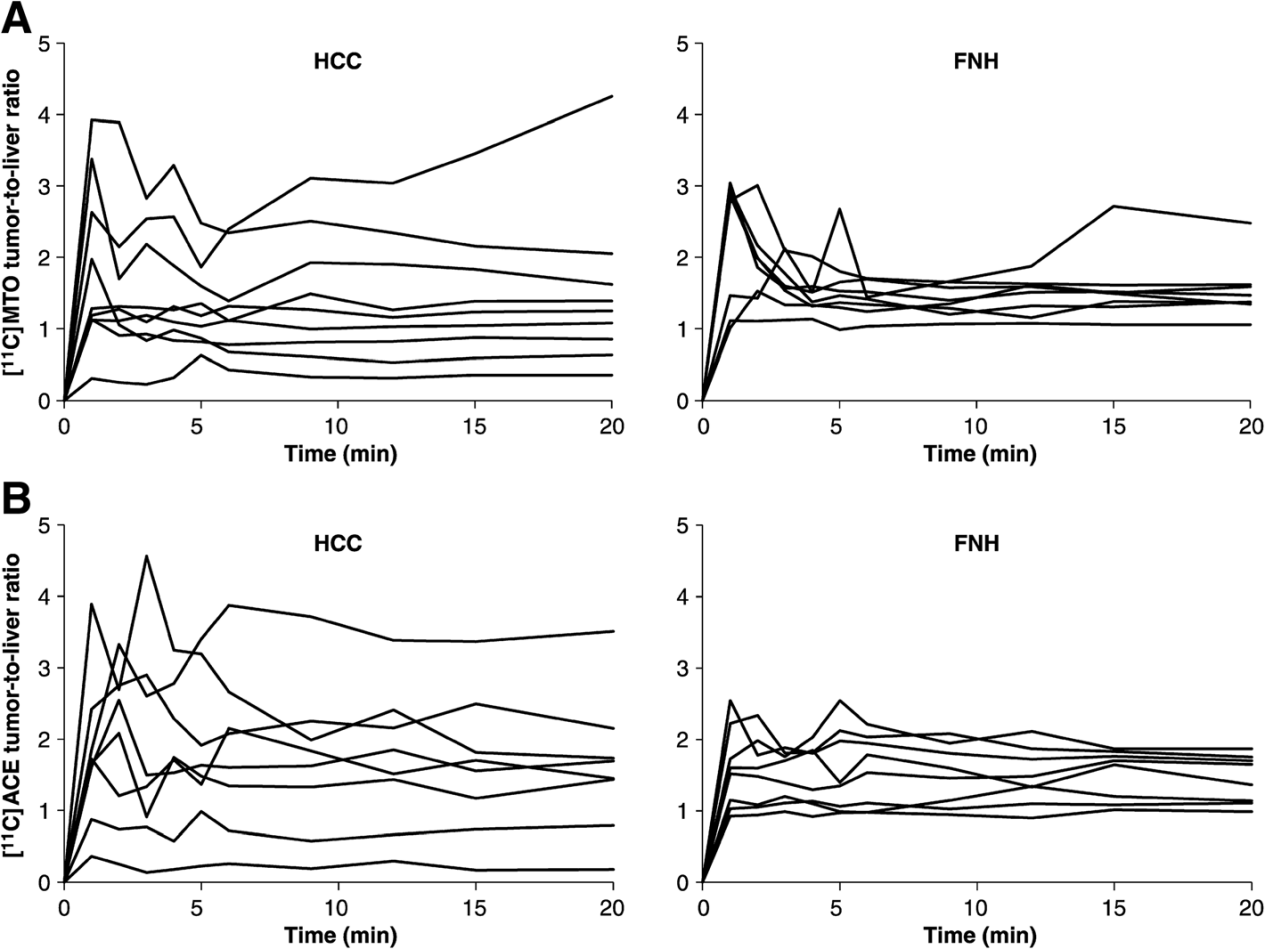
**Time-activity curves of tumors.** After injection of [^11^C]MTO (upper **A** panels) and [^11^C]ACE (lower **B** panels) expressed as tumor-to-liver ratio versus time after injection. Lines represent the mean value of individual experiments. Left panels represent hepatocellular carcinoma (HCC) and right panels focal nodular hyperplasia (FNH).

Figure 2 shows the time-activity curves of tumors after injection of [^11^C]ACE or [^11^C]MTO (*T*/*L* ratio versus time after tracer injection) for HCC (Figure 2, left panels) and FNH (Figure 2, right panels) patients. Variability of the tracer uptake (both [^11^C]ACE and [^11^C]MTO) in different tumors is clearly evident in HCC and FNH.

### Correlation of PET imaging with histopathological findings in HCC lesions

Of the 14 patients with confirmed HCC, histological assessment showed that one patient had a tumor highly differentiated, nine had moderately differentiated, and four had minimally differentiated HCCs. For highly and moderately differentiated lesions, [^11^C]MTO PET imaging was clearly positive in six of ten patients, while [^11^C]ACE PET imaging demonstrated abnormal findings in seven of ten patients. None of the patients with poorly differentiated lesions showed increased tumoral tracer uptake as compared with surrounding liver activity (*T*/*L* = 0.81 ± 0.4 with [^11^C]MTO and *T*/*L* = 0.98 ± 0.3 with [^11^C]ACE).

## Discussion

In the present study, we evaluated the feasibility of two PET tracers for the detection of HCC tumors by PET and compared it with the detection of FNH and liver metastases from other tumors. [^11^C]ACE is claimed by other authors to have high sensitivity and specificity for small nodules of HCC, and it is believed to reflect increased beta-oxidation of fatty acids. We further examined [^11^C]MTO based on reported findings of GABA receptor upregulation in HCC and binding of metomidate to GABA receptors. To our knowledge, this is the first study of [^11^C]MTO in hepatic tumors.

The [^11^C]MTO, which previously evaluated adrenal masses [20,21], demonstrated varying degrees of uptake in HCC and FNH lesions (Figure 1). The sensitivity of [^11^C]MTO for the detection of HCC and FNH was 43% and 78%, correspondingly (Table 1). The SUVs for HCC and FNH did not differ statistically (Table 2), and it was not possible to differentiate between malignant or benign lesions on the basis of visual assessments or SUV measurements. Interestingly, the whole-body [^11^C]MTO PET scan depicted some distant metastases of moderately differentiated HCC lesions. The mechanism of enhanced uptake in these lesions is intriguing but might be related to the contribution of the metastatic tumor cells to an abnormal steroidogenic pathway.

The [^11^C]ACE PET scan in 14 patients with HCC had a low detection rate. This is contradictory to two previously published studies, which reported encouraging results for the use of [^11^C]ACE in assessing HCC lesions [11-13]. In their study involving patients with histopathologically confirmed HCC, Li et al. found a sensitivity of 78% (14/18) in a patient-based analysis. Our results indicate a sensitivity of only 50% (7/14) in a patient-based analysis (Table 1). For well to moderately differentiated lesions, Li et al. report that [^11^C]ACE PET was positive in 10 of 12 patients (83%) and revealed 28 of 33 lesions (85%). In our study, [^11^C]ACE PET imaging demonstrated unexpected findings in seven of ten patients (70%) with well to moderately differentiated lesions and revealed 26 of the 46 lesions (57%). In another study by Ho et al., the detection sensitivity of [^11^C]ACE PET on a per-lesion basis in HCC patients is 87% (55 lesions, 32 patients). In our study, the detection sensitivity was only 44% (59 lesions, 14 patients) (Table 1). A possible explanation for the discrepancy between our results and the earlier findings could be the differences in races, carcinogenesis, study designs, and the relatively small number of HCC patients investigated in our study. Notably, most of the HCC in Orientals are caused by hepatitis B or hepatitis C virus infection, which are somewhat different from the hepatoma caused by alcoholism in Western people.

Approximately 30% of FNH lesions showed increased uptake of [^11^C]ACE, and it was not possible to discriminate FNH from HCC based on visual assessment or differences in semi-quantitative analysis. The results of the present study indicate that both tracers detect a variable yet low percentage of lesions representing HCC and FNH. No liver metastases of other primary tumors were detected with these tracers. However, due to the limited cases and limited kinds of cancers, i.e., only six liver metastases and four metastases of colon cancer, this might not be conclusive. The fact that liver metastases of colorectal cancer do not take up lipid PET tracers has already been published [11]. In contrast, should this group be larger, uptake would be expected in metastases from prostate cancers and some others (lung, breast).

Although CT modalities can characterize most liver lesions, MRI has emerged as the best imaging test for the detection and characterization of liver lesions. MRI is considered superior due to its high lesion-to-liver contrast and high spatial resolution, and furthermore, it does not use ionizing radiation [24]. [^18^F]FDG PET has become an established tool in the evaluation of metastatic liver disease. However, [^18^F]FDG PET has not been proven very useful for the evaluation of HCC. Several studies have revealed that the sensitivity of [^18^F]FDG PET in the diagnosis of HCC is approximately 50% [25,26]. Although other positron-emitting tracers ([^11^C]ACE, [^11^C]choline, [^18^F]fluorocholine, and [^18^F]fluorodeoxygalactose) have been evaluated for diagnosis of HCC as well, none of them succeeds fully alone [11-13,17,18,27]. For example, [^11^C]choline has the same pitfalls but somewhat better sensitivity values. However, [^18^F]fluorocholine has been registered in several EU countries in this indication, and the dual-tracer PET/CT (i.e., [^18^F]FDG and [^11^C]ACE or [^18^F]FDG and [^18^F]fluorocholine) has shown good results for HCC detection and staging [18,19].

### Limitations

It is internationally agreed that the medical challenge is to detect HCC in the early phase when tumors are less than 2 cm because then treatment can be curative. In this study population, the HCC tumor sizes ranged from 3 to 19 cm in diameter, as determined by MRI. This is reasonable for the purpose of testing the feasibility of the two tracers, but lacks any evidence for the detection of smaller tumors. The number of patients included in this study (14 HCC, 9 FNH, and 10 other hepatic tumors) is limited, which can be regarded as a study limitation. Accordingly, the results of sensitivity and specificity should be considered as approximates.

The most intensive liver hotspots were used for the PET analysis. Unfortunately, these lesions were not always the same as those that were biopsied. We recognize that due to tumor heterogeneity, the observed SUV values do not necessarily represent the histological samples. Patients are treated according to histological findings, and it is not possible to take biopsies from all the liver lesions.

## Conclusions

None of the secondary liver metastases were PET-positive for any of the two tracers. None of the tracers, however, reached a level of sensitivity for detection of hepatic tumors that would support their appropriateness as diagnostic PET tracers for HCC.

## Abbreviations

CT: Computed tomography; [11C]ACE: [^11^C]acetate; [11C]MTO: [^11^C]metomidate; [18F]FDG: 2-[^18^F]-fluoro-2-deoxy-*D*-glucose; FNH: Focal nodular hyperplasia; GABA: Gamma-aminobutyric acid; HCC: Hepatocellular carcinoma; MR: Magnetic resonance imaging; PET: Positron emission tomography; ROI: Region of interest; SUVmax: Maximum standardized uptake value; SUVmean: Mean standardized uptake value; T/L: Tumor-to-liver ratio; US: Ultrasound

## Competing interests

The authors declare that they have no competing interests.

## Authors’ contributions

AR participated in analyzing and interpreting the data, drafting and revising the manuscript and enhancing its intellectual content, obtaining financing for the study. AN participated in acquiring, analyzing, and interpreting the data, drafting the manuscript. HN participated in recruiting the patients; acquiring, analyzing, and interpreting the data; and writing of the manuscript. RL and HN participated in recruiting the patients, drafting the manuscript. KN participated in providing the [^11^C]MTO and [^11^C]acetate tracers and drafting the manuscript. RP and JV participated in interpreting the MRI data and drafting the manuscript. MK and HK participated in performing the histopathological analysis and drafting the manuscript. JO participated in acquiring the data and drafting the manuscript. PR participated in interpreting the data and revising the manuscript. MS contributed to the study concept and design and participated in acquiring, analyzing, and interpreting the data and revising the manuscript. All authors read and approved the final manuscript.
